# Physicochemical, cytotoxicity and *in vivo* biocompatibility of a high-plasticity calcium-silicate based material

**DOI:** 10.1038/s41598-019-40365-4

**Published:** 2019-03-08

**Authors:** Cláudio M. A. Ferreira, Luciana M. Sassone, Alexia S. Gonçalves, Jorge José de Carvalho, Christopher J. Tomás-Catalá, David García-Bernal, Ricardo E. Oñate-Sánchez, Francisco J. Rodríguez-Lozano, Emmanuel João Nogueira Leal Silva

**Affiliations:** 1 Endodontic Department, School of Dentistry, Rio de Janeiro State University, Rio de Janeiro, Brazil; 2 Department of Histology and Embryology, Laboratory of Ultrastructure and Tecidual Biology, Biomedical Center, Rio de Janeiro State University, Rio de Janeiro, Brazil; 3 Cell Therapy Unit at Hospital Clínico Universitario Virgen de la Arrixaca, Biomedical Research Institute of Murcia, University of Murcia, Murcia, Spain; 4 Department of Special Care in Dentistry and Gerodontology, University of Murcia, Murcia, Spain

## Abstract

The purpose of this work was to evaluate the physicochemical properties, the cytotoxicity and *in vivo* biocompatibility of MTA Repair HP (MTA HP) and White MTA (WMTA). The setting time, flow, radiopacity and water solubility were assessed. To the cytotoxicity assay, primary human osteoblast cells were exposed to several dilutions of both materials eluates. MTT assay, apoptosis assay and cell adhesion assay were performed. The *in vivo* biocompatibility was evaluated through histological analysis using different staining techniques. No differences were observed between MTA HP and WMTA for setting time, radiopacity, solubility and water absorption (P > 0.05). However, MTA HP showed a significantly higher flow when compared to WMTA (P < 0.05). Cell viability results revealed that the extracts of WMTA and MTA HP promoted the viability of osteoblasts. After incubation of cells with the endodontic cement extracts, the percentage of apoptotic or necrotic cells was very low (<3%). Furthermore, SEM results showed a high degree of cell proliferation and adhesion on both groups. MTA HP showed similar *in vivo* biocompatibility to the WMTA and the control group in all time-points. The MTA HP presented adequate physicochemical and biological properties with improved flow ability when compared to WMTA. Such improved flow ability may be a result of the addition of a plasticizing agent and should be related to an improvement in the handling of MTA HP.

## Introduction

The materials used for perforation repair and root-end filling should be biocompatible and bioactive because they are in close contact with surrounding tissues^1^. The presence of stem cells in periapical tissues and their interaction with endodontic materials have been considered important in order to favor healing^2^. Thus, the biocompatibility, excellent sealing ability, hard tissue induction and conduction, and high rate of success have made bioactive endodontic cements a good choice for root-end filling^3,4^.

Mineral trioxide aggregate (MTA) is considerable the gold standard material for several clinical procedures, as confirmed by a robust body of evidence supporting the physicochemical and biological properties of this material^3,4^. However, the traditional MTA formulation presents some drawbacks such as handling difficulties, granular consistency and long setting-time^5^. Furthermore, the use of bismuth oxide as a radiopacifying agent may promote discoloration of the tooth and marginal gingiva^5,6^ and affect the physicochemical properties of this material^5,7^.

In the search for the improvement of the MTA, a new formulation of the White MTA (Angelus, Londrina, PR, Brazil) called MTA Repair HP (MTA “High Plasticity”, Angelus) was launched in the form of a bioceramic material with high plasticity aiming to maintain the biological properties of the MTA, improving its chemical and physical properties. The main differences to its predecessor are the addition of an organic plasticizer to the distilled water (promoting greater plasticity to the material) and the exchange of bismuth oxide by calcium tungstate as a radiopacifying agent. Studies demonstrated that the use of plasticizers may lead to improvements in the physicochemical properties and handling of the MTA^8,9^. Moreover, the substitution of bismuth oxide by calcium tungstate avoid tooth staining^5,6^ and reduce interferences in physicochemical properties^10^. Also, calcium tungstate has been shown to be more biocompatible than bismuth oxide^11^.

An improvement in the mechanical properties of the MTA Repair HP was demonstrated in a study evaluating the resistance to dislodgment, in which MTA Repair HP outperformed White MTA^12^. In addition, recent studies have demonstrated the maintenance of the acclaimed biological properties of the White MTA by its successor, MTA Repair HP^13–15^. The presentation of this new material seems to be innovative and up to now encouraging results has been published^12–15^. However, the studies are limited to performing histopathological analysis of the inflammatory reaction of the material^13^. Wound healing involves complex interactions among inflammatory mediators and cells which angiogenesis and tissue remodeling play an important role in this process, being necessary to evaluate these aspects^16^. Moreover, previously published cell culture studies were only performed using fibroblasts^13^ or human dental pulp stem cells^14,15^. It is necessary to evaluate the reaction of this new material in a cell culture closed to the clinical application, such as periodontal ligament stem cells and osteoblasts. In addition, studies comparing the MTA HP and its predecessor from the physicochemical point of view have not been performed. Thus, the aim of this study was to evaluate the physicochemical properties, *in vitro* cytotoxicity and *in vivo* biocompatibility of MTA Repair HP and compare the results with those obtained by its predecessor White MTA. The physicochemical properties were assessed through the evaluation of setting time, flow, radiopacity and water solubility. The cytotoxicity of the tested materials was tested using MTT assay, apoptosis assay and cell adhesion. The biocompatibility was evaluated through histological analysis using different staining techniques, including hematoxylin-eosin, picrosirius red, Weigert, Gomori´s trichome and immunohistochemical staining for VEGF markers. The null hypotheses tested in this study were that the new formulation of MTA HP would have no differences on: (i) physicochemical properties, (ii) cytotoxicity and (iii) biocompatibility, when compared to White MTA.

## Results

### Physicochemical properties

The mean and standard deviation of flow, setting time, radiopacity, solubility and water absorption are shown in Table 1. No differences were observed between MTA Repair HP and White MTA for setting time (p = 0.0921), radiopacity (p = 0.1198), solubility (p = 0.8538) and water absorption (p = 0.2856). However, MTA Repair HP showed a significantly higher flow when compared to White MTA (p = 0.0001).

**Table 1:** The mean and standard deviation (SD) of flow (mm), setting time (min), radiopacity (mm Al), water absorption (%μg/mm³) and solubility (%μg/mm³) of MTA HP Repair and White MTA.

	MTA Repair HP	White MTA	P value
Flow (mm)	16.4 ± 0.8^A^	9.5 ± 1.4^B^	0.0001
Setting time (min)	22.2 ± 2.3^A^	17.8 ± 2.1^A^	0.0921
Radiopacity (mm Al)	4.44 ± 0.4^A^	4.86 ± 0.3^A^	0.1198
Solubility (%μg/mm³)	−2.46 ± 0.9^A^	−2.20 ± 0.4^A^	0.8538
Water absorption (%μg/mm³)	10.84 ± 1.7^A^	13.56 ± 2.3^A^	0.2856

Different superscript letters represent statistical differences (P < 0.05).

### Cytotoxicity assays

#### MTT assay

**Figure 1:**
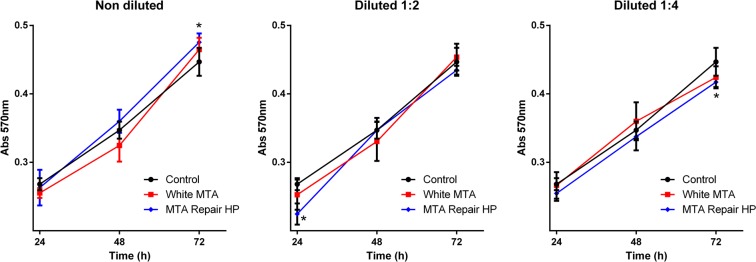
Determination of osteoblast viability after treatment with the different endodontic extracts for the indicated times by MTT assay. Values were significantly different compared to the control group (*p < 0.05; **p < 0.01; ***p < 0.001, respectively; ANOVA and Tukey’s post-hoc test).

Results presented in Fig. 1 indicate the effects of MTA Repair HP and White MTA extracts on primary osteoblast viability. Extracts of theses endodontic cements exhibited similar rates of cell viability compared to control. Interestingly, at 72 hours there was a small but significant increase in the viability of the cells grown in presence of undiluted MTA Repair HP (p = 0.045), whereas MTA Repair HP 1:4 produced a statistically significant reduction in cellular viability after 72 hours (p = 0.016).

#### Apoptosis/necrosis of osteoblasts hPDLSCs delete hPDLSCs in presence of eluates

**Figure 2:**
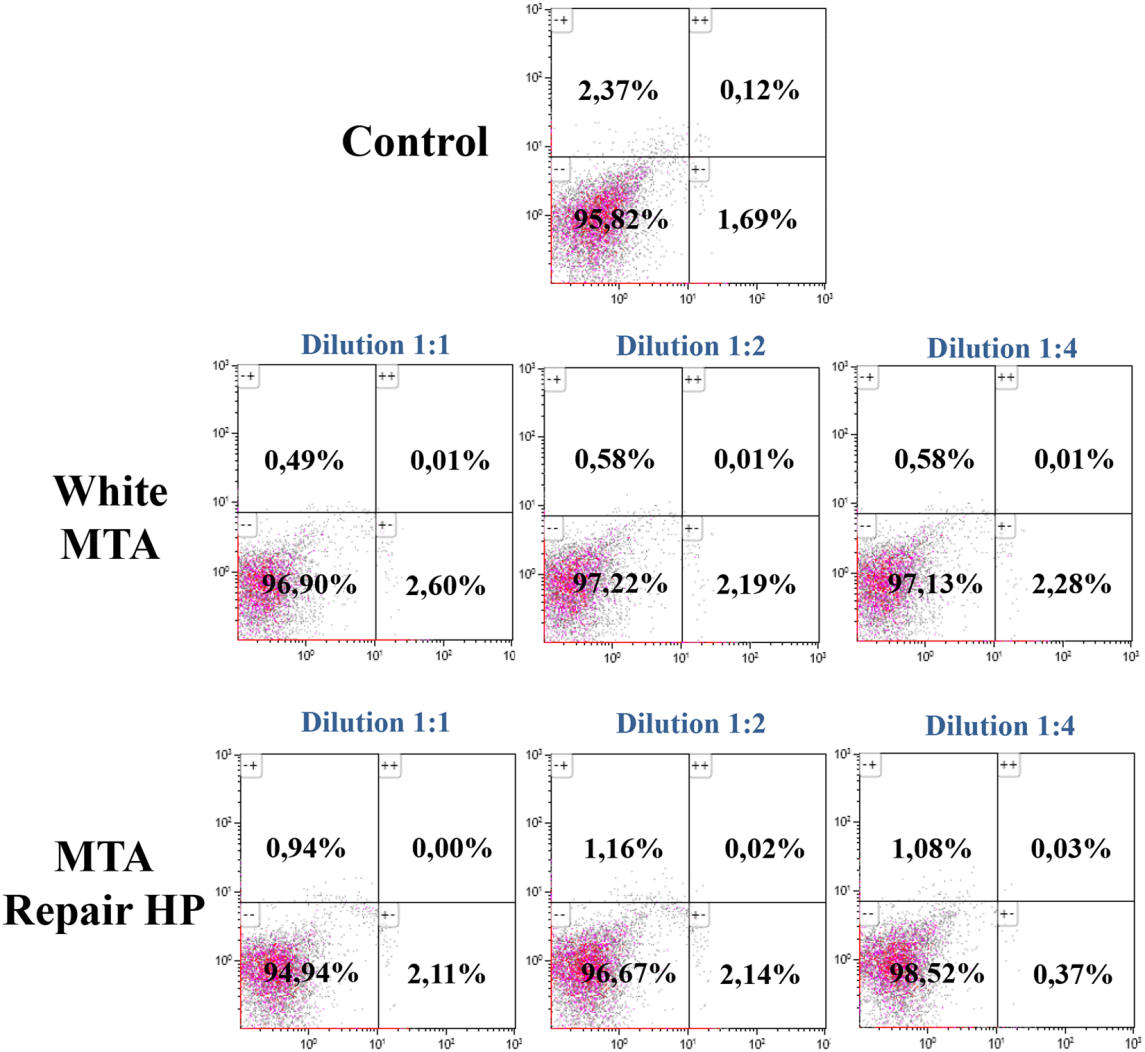
Analysis of induction of apoptosis of the different endodontic extracts on primary osteoblast by flow cytometry. Osteoblast were incubated with the different extracts for 72 h, followed by staining with Annexin-V-FITC and 7-AAD. Cells cultured with osteoblast basal medium were used as control. Numbers inside dot plots represent percentage of live (bottom left quadrant), early apoptotic (bottom right quadrant) and late apoptotic/necrotic cells (left and right upper quadrants). Flow cytometry data show representative results from one of three independent experiments.

As shown in Fig. 2, apoptosis analysis by using Annexin-V Apoptosis Detection Kit was performed to discard possible cytotoxic effects in untreated primary osteoblasts, or after incubation to the studied endodontic cements eluates at different concentrations. Undiluted extracts of White MTA and MTA HP was associated with more than 94% of viable cells after 72 hours.

#### Cell attachment on materials

**Figure 3:**
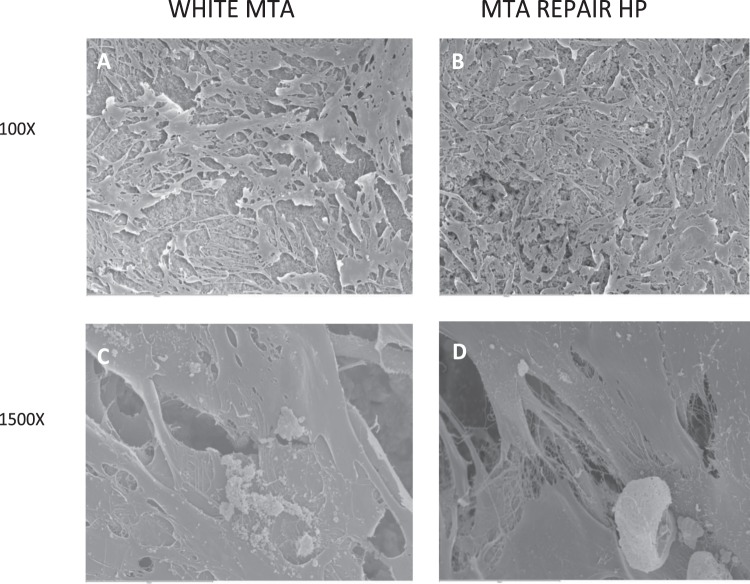
SEM microphotographs of osteoblasts cultured into MTA Repair HP and White MTA specimens. Scanning electron microscopic morphology images of osteoblasts *h*PDLSCs please delete hPDLSCs attached to White MTA (**A**,**C**) and MTA Repair HP (**B**,**D**) surfaces were obtained at 72 hours.

As shown in Fig. 3, cell adhesion to all materials surface was analyzed after 72 hours of culture. Using SEM analysis at high magnifications (1500X), extending cytoplasmic processes and filopodia were observed in both materials, which enabled the anchorage of cells. At 100X magnification, flattened cells with multiple prolongations proliferated on the uneven surface and in the pores of the granules to create bridges across the particles, organizing a multilayer covering the material surfaces.

### *In vivo* Biocompatibility

The results for the histologic examination are shown in Table 2.

**Table 2:** Results of evaluations of the inflammatory reaction, fibrillar content of the extracellular matrix, and immunoreactivity to VEGF.

Time	Groups	Scores (%)				Median	*n*	Collagen fibers (%)		Capsule	Elastic fibers	VEGF
		1	2	3	4			Type I	Type III			
7 days	*Control* ^*A*^	0	0	60	40	3	10	21	79	Thick	+	++
	*White MTA* ^*A*^	0	20	60	20	3		56	44	Thick	++	+++
	*MTA Repair HP* ^*A*^	0	20	40	40	3		36	64	Thick	+	++
30 days	*Control* ^*A*^	20	60	20	0	2	10	37	63	Thin	+	+
	*White MTA* ^*A*^	20	60	20	0	2		58	42	Thin	+	+
	*MTA Repair HP* ^*A*^	0	80	20	0	2		54	46	Thin	+	+
60 days	*Control* ^*A*^	80	20	0	0	1	10	46	54	Thin	+	+
	*White MTA* ^*A*^	40	60	0	0	1		79	21	Thin	+	+
	*MTA Repair HP* ^*A*^	40	40	20	0	2		85	15	Thin	+	+

*Scores: a semiquantitative analysis was performed which same letters indicate no statistical difference among the groups (P = 0.4284 for 7 days, P = 0.6998 for 30 days, and P = 0.1048 for 60 days). A qualitative analysis was performed in the other histological evaluations. Collagen fibers: expressed as a percentage of type I and type III collagen fibers. Presence of elastic fibers: categorized in few to rare (+); moderate (++) and intense (+++). Intensity of VEGF immunoblotting: classified in weak (+); moderate (++) and intense (+++).

**Figure 4:**
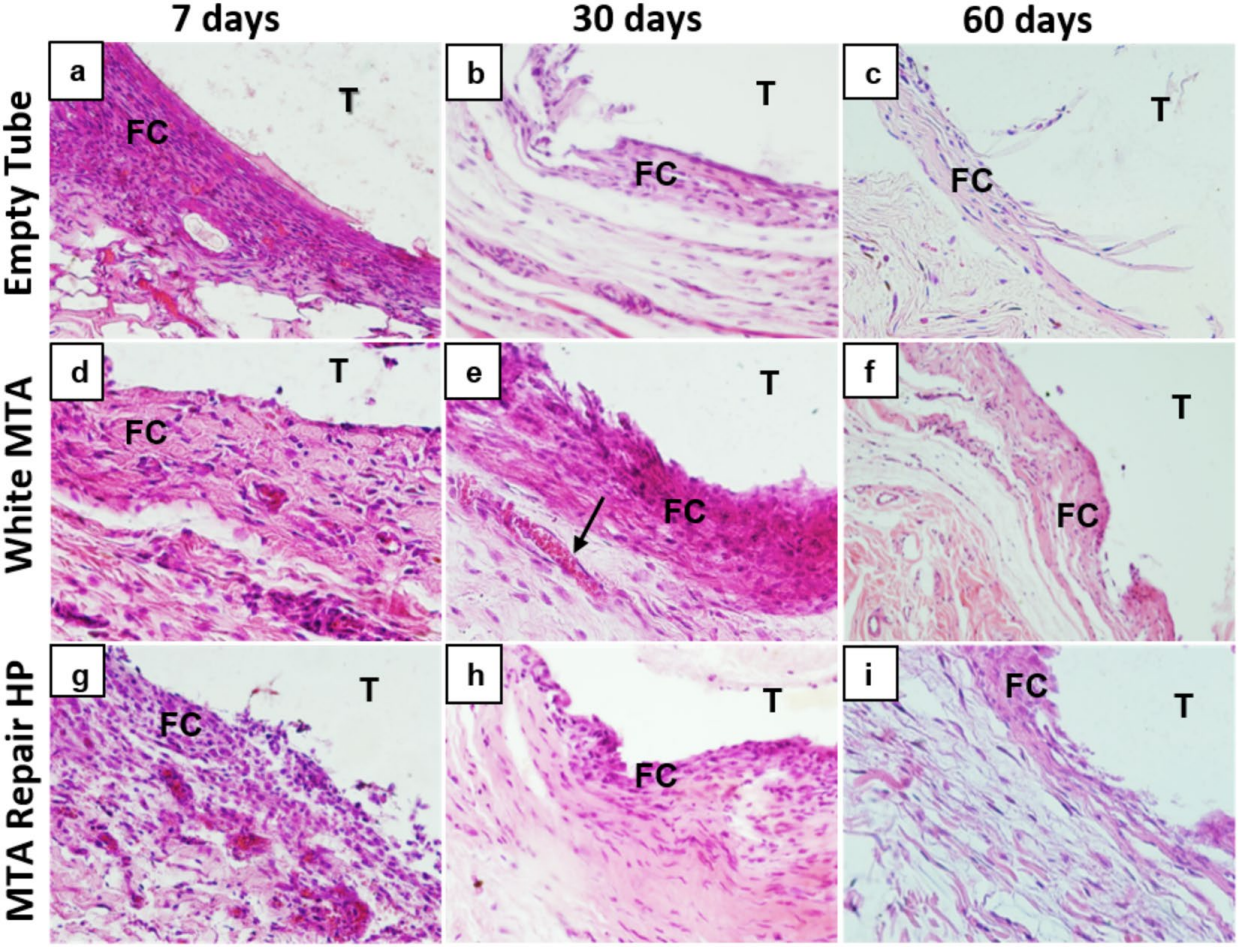
Representative images of subcutaneous tissue reactions in the experimental groups stained with Hematoxylin and Eosin. (**a**), (**d**), and (**g**) - presence of intense inflammatory infiltrate, thick fibrous capsule (FC) adjacent to the implanted tube lumen (T). (**b**), (**e**), and (**h**) - presence of moderate inflammatory infiltrate in the White MTA and MTA Repair HP groups, mild inflammatory infiltrate in the control group (empty tube), thin fibrous capsule (FC) in the control and MTA Repair HP groups, thick fibrous capsule in the White MTA group and congested capillary (arrow). (**c**), (**f**) and (**i**) - presence of mild to absent inflammatory infiltrate and thin fibrous capsule (FC) in all groups. Original magnification 400x.

**Figure 5:**
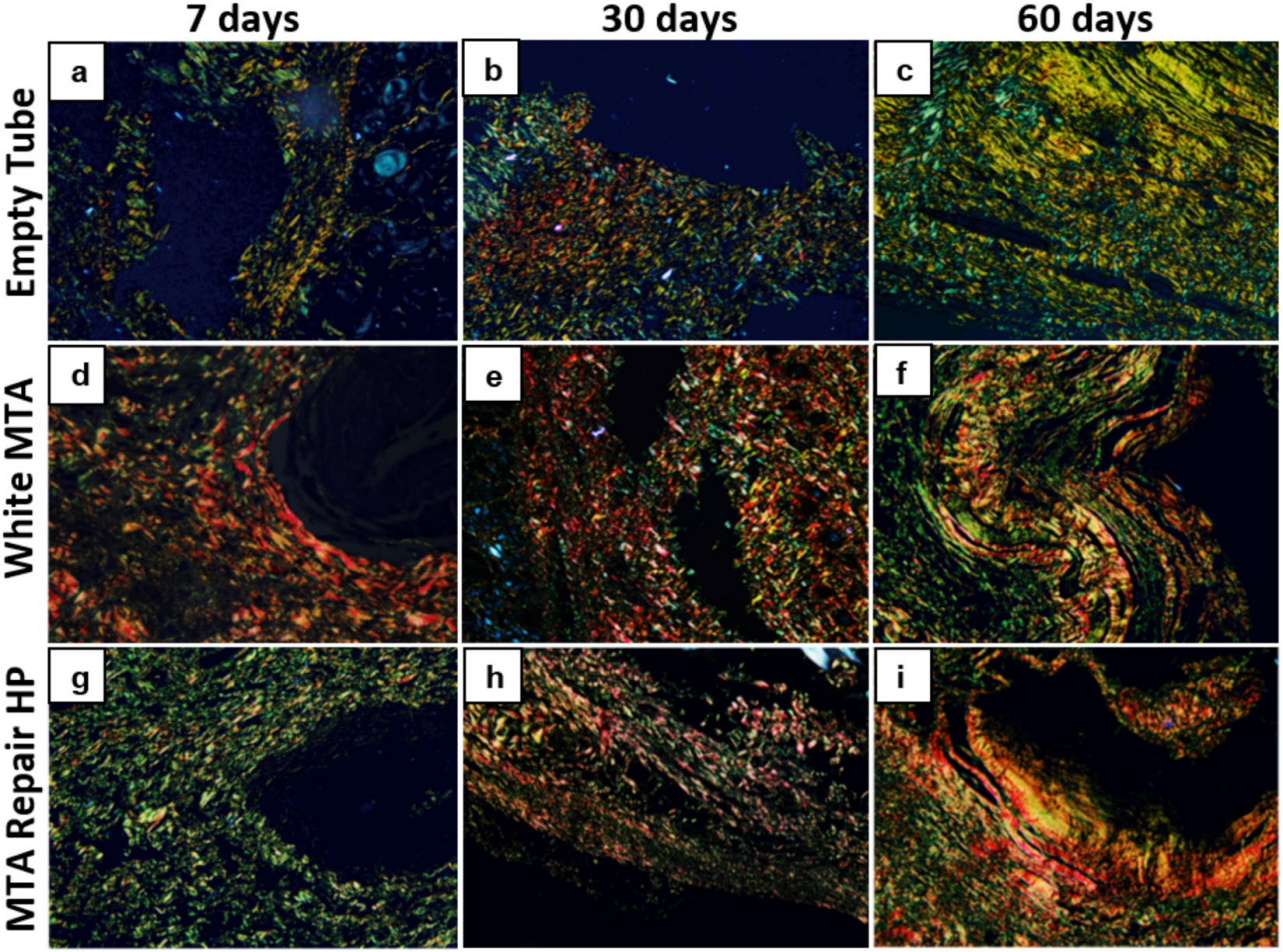
Representative images of subcutaneous tissue reactions in the experimental groups stained with picrosirius red. Type I collagen fibers visualized in red and yellow colors and type III collagen fibers visualized in green color; (**a**), (**d**) and (**g**) - predominance of type I fibers in White MTA group; predominance of type III fibers in the control (empty tube) and MTA Repair HP groups. (**b**), (**e**) and (**h**) - predominance of type I fibers in White MTA and MTA Repair HP groups, predominance of type III fibers in the control group. (**c**), (**f**) and (**i**) - predominance of type I fibers in White MTA and MTA Repair HP groups, slight predominance of type III fibers in the control group. Original magnification 200x.

At 7 days, most specimens in all the groups showed moderate to severe inflammation, with intense infiltrate of inflammatory cells consisting of lymphocytes, macrophages and plasmocytes (Fig. 4 and Supplementary Fig. S2); at this time, the fibrous capsule in the tube opening region was thick (Table 2 and Fig. 4). In the White MTA group an intense immunostaining was observed in endothelial cells of capillaries, arterioles and venules. Some vascular muscle cells and inflammatory cells (macrophages) also showed VEGF immunoreactivity. The immunostaining in the other groups were moderate (Table 2 and Supplementary Fig. S1). In comparison with the other groups, the elastic fibers had a small increase in the connective tissue near the muscular layer in the White MTA group (Table 2 and Supplementary Fig. S2). The presence of type I collagen fibers was higher than type III in the White MTA group whereas the reverse was observed in the control and MTA Repair HP groups (Table 2 and Fig. 5).

At 30 days, all groups showed a reduction in the number of inflammatory cells, congested blood vessels and capillaries as well as in the thickness of the fibrous capsules that surrounded the tubes, although some samples were still observed in the White MTA and MTA Repair HP groups, with thick capsule (Table 2 and Fig. 4). At this point all groups showed a weak immunostaining and from few to rare elastic fibers, without difference between the groups (Table 2). An increase of a proportion of type I collagen fibers and a decrease of type III collagen fibers was observed in all groups, although control group still presented a predominance of type III collagen fibers (Table 2 and Fig. 5). It was also observed an increase of the thickness and a greater organization of the collagen fibers in the connective tissue (Supplementary Fig. S2).

At 60 days all groups showed from rare to moderate inflammatory cells dispersed in connective tissue with lymphocyte prevalence and presence of giant cells without differences among the control and experimental groups (p = 0.1048) (Table 2 and Fig. 4). All groups presented mild to absent inflammatory infiltrate with the presence of rare dispersed inflammatory cells (plasma cells and lymphocytes) arranged in connective tissue and few blood vessels (Supplementary Fig. S2). At this time, the fibrous capsule in the tube-opening region was thin in the all groups (Fig. 4). The results of immunostaining and the presence of elastic fibers did not change since the 30 days period. An increase and a predominance of type I collagen fibers was observed in White MTA and MTA Repair HP groups, while the control group still presented a predominance of type III collagen fibers (Table 2 and Fig. 5).

## Discussion

Cements used for retrograde filling in endodontic microsurgery come into direct contact with periapical tissues. Deposition of cementum on the cut root face is considered a prerequisite for healing. Physical seal of the root-end filling and cementum deposition create a double seal^17^. As pointed out in the Introduction section, MTA HP has previously been evaluated for biomineralization^13^, biocompatibility using hematoxylin and eosin staining^13^, and cytotoxicity of L929 fibroblasts^13^ and human dental pulp stem cells^14,15^. However, to the best of the authors’ knowledge, this is the first time that physicochemical properties and different aspects of inflammation such as the fibrillary composition of the extracellular matrix and the presence of angiogenesis were evaluated. In addition, a cell line close to the clinical situation, such as osteoblasts was also evaluated. In addition, the reference material chosen in the present study is its predecessor, which makes this assessment more reliable from a scientific point of view.

The present study evaluated some of the main physicochemical properties that should be considered for a suitable endodontic cement. The results of the present study demonstrated that MTA Repair HP and its predecessor White MTA presented similar results in the setting time, radiopacity, solubility and water absorption tests, all in accordance with ISO specifications. In the present study, ISO 4049 were used to test water solubility and water absorption. This ISO 4049 was selected as it includes test procedures for both solubility and water absorption, allowing determining different physicochemical aspects using the same samples. In contrast, ISO 6876 only includes the solubility test and the readings calculated from solution, do not take into consideration the evaporation rate of the relative humidity which can interfere directly in the results, as previously reported by Cutajar *et al*.^18^.

However, MTA Repair HP showed higher flow ability when compared to White MTA. Therefore, the first null hypothesis was rejected. This difference can be directly attributed to the presence of a plasticizer contained in the mixing liquid of MTA Repair HP. During material manipulation it is possible to observe that MTA Repair HP present a creamy and homogeneous characteristic while White MTA have a coarse sandy consistency. Considering the importance of the ideal flow ability that endodontic cements should present reducing the difficulty of handling^19^, it is possible to affirm that the greater flow ability presented by MTA Repair HP can be considered an advantage when compared to White MTA. Although no previous study evaluated the influence of flow on the wettability or adhesion of a root repair material, such as MTA HP, a recent study^12^ demonstrated that MTA HP demonstrated higher push-out bond strength values when compared to its predecessor, White MTA. According to the authors, one of the aspects that might influence this better result of MTA HP is the better handling property of this new material. However, it is important that future studies be performed evaluating this influence on wettability and adhesion properties.

Regarding cytotoxicity, we analyzed the biological cell responses of human osteoblasts with respect to cell viability, apoptosis and morphology in the presence of these cements. Osteoblasts were used in the present study because this cell type has important advantages such as being easily obtained and allows to test the mechanism of action of new biomaterials using the patient´s own cells, providing unprecedented human models^20,21^.

The cytotoxicity results suggested that both materials, MTA Repair HP and White MTA, exhibited an effect comparable to that of the control. For this reason, the second null hypothesis was accepted. In agreement with our results, previous studies^14,15^ found that MTA Repair HP and White MTA promoted adequate biological effects on hDPSCs in terms of cell proliferation, morphology, migration and attachment. Cell adhesion is another appropriate indicator of cytocompatibility since this process plays a crucial role during the perirradicular reparation^22^. In this study, the adhesion and morphology of osteoblasts on cement disks were evaluated using a scanning electron microscope (SEM). At 72 hours, a large number of flattened and spreading cells were seen to have covered the surface of disks. Other authors have reported similar cell degrees of attachment using MTA-based materials^14,15,23^.

It should be noted that *in vitro* cytotoxicity assays comprise the first level of biocompatibility analysis of a material and that it may undergo influences, depending, for example, on the cell type used. Compensating for the deficiencies of an *in vitro* model, subcutaneous tissue tests was also performed. In the present study the results of the *in vivo* model complements those obtained in the *in vitro* model. Overall, the *in vivo* biocompatibility results demonstrated a moderate to severe inflammatory reaction at 7 days. This reaction decreased over time in both materials tested suggesting that the tested materials were biocompatible. Thus, the third null hypothesis was also accepted. These results are in accordance with recent literature in which no difference in *in vivo* biocompatibility was observed between MTA Repair HP and White MTA after 7 and 30 days^13^. The initial inflammatory reaction may be caused by the surgical trauma produced during tube placement, in addition to the toxic effects of the implanted materials. Moreover, it is normal to observe an initial inflammatory reaction when using calcium-silicate based cements, which can be explained mainly by the alkalinity damage provoked on adjacent tissues^13,24^.

In addition to the hematoxylin-eosin staining, other staining techniques were performed in order to better understand the behavior of the tested materials. VEGF is a powerful mitogen for endothelial cells and induces angiogenesis, because it inhibits the apoptosis of these cells, and promotes the migration of endothelial precursors^25^. The higher immunoreactivity for VEGF in all groups at seven days confirms the greater presence of vascular congestion, perivascular inflammatory infiltrate and blood extravasation. Gomori’s trichrome staining allowed visualizing an increase of the thickness and a greater organization of the collagen fibers in the connective tissue over time^26^, evidencing tissue repair along the time.

During the remodeling process of the extracellular matrix, there is a greater predominance of type III collagen in the initial repair phase, with less thickness and less birefringence than type I collagen^27^. The results of the Picrosirius Red staining showed tissue healing over time with an increase in the amount of type I collagen fibers and a decrease in type III fibers in all tested groups. However, a higher prevalence of type III collagen was observed in all evaluation periods of the control group and at 7 days at MTA Repair HP. As in the initial periods there were no differences in the intensity of inflammatory reaction between the groups (as demonstrated in the HE and Gomori’s trichrome staining), it is suggested that the tested materials can stimulate the cytokines and the release of growth factor by the host cells. This can promote a faster maturation of the scar tissue when compared to the control group^16^. According to the results of the Weigert and Picrosirius Red stains, the White MTA can stimulate extracellular matrix remodeling more effectively in the initial healing period when compared to MTA Repair HP.

The similar results obtained using MTA Repair HP and White MTA indicate that the new formulation did not alter the biocompatibility of the predecessor cement. It has been previously demonstrated that, the addition of plasticizers, such as propylene glycol, to MTA showed good biological response and no interference in tissue repair^9^. Moreover, the calcium tungstate also demonstrated adequate cytocompatibility in contact with periodontal and osteogenic cells^11^. Both repair cements presented good tolerance for the connective tissues compared to the control group. However, in the initial period White MTA group presented a higher immunoreactivity for VEGF and a higher concentration of elastic fibers and type I collagen fibers in comparison with other groups, suggesting a more effective tissue remodeling. Regarding immunoreactivity to VEGF, all groups presented an intense immunostaining in the initial period of evaluation and a decrease over time. These findings may be correlated with the decrease of angiogenesis that occurs according to the maturation of the extracellular matrix and the decrease of the inflammatory process.

Taken together, the present study demonstrated that the MTA Repair HP presented adequate physicochemical and biological properties with improved flow ability when compared to White MTA. Such improved flow ability may be a result of the addition of a plasticizing agent that should be related to an improvement in the handling of MTA Repair HP. The biocompatibility of both materials might have direct relation with materials composition and with some properties such as the low solubility and the short setting time, avoiding leakage of material to perirradicular tissues.

## Materials and Methods

MTA Repair HP and White MTA were mixed according to the manufacturer’s instructions for all the tests. The composition of the evaluated materials can be found as Supplementary Table S1.

### Physicochemical properties

Sample size used for the physicochemical properties was determined by the selected ISO.

#### Flow test

The flow test was performed following ISO 6876/2012 specifications^28^. Five samples were used for each cement. A final volume of 0.05 mL cement was prepared and centrally placed on a glass plate. After 180 seconds, a second glass plate was carefully and centrally placed on top of the cement followed by a 100 g weight to make a total mass of 120 g. Ten minutes after the onset of mixing the weight was removed, and the maximum and minimum diameters of the compressed cement were measured with a digital caliper. The mean of 3 measurements for each sample, expressed to the nearest millimeter, was taken to obtain the flow rates.

#### Radiopacity test

The radiopacity test was performed following ISO 6876/2012 specifications^28^. Cylindric samples from each material were manufactured by pouring the manipulated cements into metallic rings measuring 10 mm in diameter and 1 mm in thickness (n = 5). The filled rings were kept at 37 °C until the cements were completely set. The specimens were then removed, and the thickness was checked with a digital caliper (700–126; Mitutoyo MTI Corp, Tokyo, Japan). All cements were placed on 5 occlusal films (Insight; Kodak Company, Rochester, NY) along with an aluminum step wedge graduated from 1 to 10 mm Al (in 1-mm increments). Radiographs were taken by using a radiographic unit (XR 6010; Gnatus, Ribeirão Preto, SP, Brazil) operating at 70 kV and 10 mA, with the exposure set at 0.3 seconds and a focus-film distance of 30 cm. After processing, the radiopacity value was determined according to the radiographic density, which was also converted into millimeters of aluminum. Conversion was performed as described previously^29^.

#### Setting time test

The setting time test was performed according to the ISO 6876/2012 specification^28^. Discs samples of the materials, of 10 mm in diameter and 1 mm thickness were prepared (n = 3). A Gilmore-type needle with a weight of 100 ± 0.05 g and with a flat end of 2.0 ± 0.1 mm in diameter was used to determine the initial setting time. The Gilmore apparatus was placed in a well-sealed plastic box at 37 °C and 95% relative humidity. The methodology used was as follow: 180 s after mixing, measures were taken every 60 s until the setting time was approached. The setting time was defined as the time point at which the indenter failed to leave a definite mark on the surface of the samples. The test was performed in triplicate.

#### Water Solubility and Water absorption tests

The water solubility (WSL) and water absorption (WSR) was performed following ISO 4049:2009 specification^30^. Ten specimens of each material were prepared using a metal mold (1 mm thick and 6 mm in diameter). The specimens were weighed after 24 h until a constant initial mass (m1) was obtained; after one week of storage in distilled water (1 ml for each specimen) at 37 °C (m2); and until a constant final mass (m3) after removal from the solution. The water solubility (WSL = [(m1 − m3)/m3] × 100) and sorption (WSR = [(m2 − m3)/m3] × 100) were calculated as percentages of the original weight.

### Cytotoxicity assays

Sample size used for the cytotoxicity assays was determined by the selected ISO.

#### Endodontic cement extracts

Under aseptic conditions, MTA Repair HP and White MTA were mixed according to the manufacturers’ instructions, placed in sterile cylindrical molds of 2-mm height and 5-mm diameter and stored inside a dark container at 37 °C for 48 hours to allow complete setting (n = 30). After this time period, samples disks were stored in culture medium (DMEM) for 24 hours at 37 °C in a humid atmosphere containing 5% CO_2_. This procedure was carried out according to International Organization for Standardization (ISO) guideline 10993-12 and the ratio of the specimen surface area was 1.5 cm^2^/mL (ISO 10993-5)^31,32^. Extracts obtained were filtered, diluted (undiluted, 1/2, 1/4) and used in the subsequent experiments.

#### Isolation and culture of NHOst

NHOst human bone primary osteoblasts were provided by Lonza (Basel, Switzerland). Cells were seeded in 75-cm^2^ culture flasks (Corning, New York, USA) in Osteoblasts Basal Medium and Growth Medium Supplement Mix (both from PromoCell, Heidelberg, Germany) and cultured at 37 °C in a 5% CO_2_ atmosphere. The culture medium was replaced every 2–3 days.

#### MTT assay

The metabolic activity of NHOst seeded with endodontic cements extracts was determined by 3-(4,5-dimethyl-thiazoyl)-2,5-diphenyltetrazolium bromide (MTT) method. Briefly, 1 × 10^3^ cells were seeded in 96-well plates with 180 μL of culture medium for 24 h. Material extracts were then added, and cells incubated at 37 °C in a 5% CO_2_ atmosphere for 24, 48 or 72 hours., At the indicated time points, the samples were incubated with 1 mg/mL of MTT for 4 h. Then, the MTT was removed and the formazan crystals were dissolved with dimethyl sulfoxide for 30 min. Finally, absorbance values were read at 570 nm (Abs570) by using an automatic microplate reader (ELx800; Bio-Tek Instruments, Winooski, VT, USA). Cells cultured without extracts were used as negative control. The MTT assay was performed in three independent experiments, with three replicate wells for each experimental point.

#### Apoptosis/necrosis assay

Induction of apoptosis of the different endodontic extracts on primary osteoblasts after 72 h of culture was evaluated after staining with Annexin-V-FITC and 7-AAD. Briefly, cells were resuspended in 100 μl of 1x Annexin-V binding buffer, and incubated with 5 µl of Annexin V-FITC and 5 µl 7-AAD at room temperature for 15 min. Finally, 400 μl of same binding buffer were added, and analyzed in a BD FACSCanto II flow cytometer (BD Biosciences, San Jose, CA, USA) to calculate percentages of live, early apoptotic and late apoptotic/necrotic cells.

#### Scanning electronic microscopy (SEM)

Different samples of White MTA and MTA Repair HP were shaped into 1.6-mm thick disks of 5 mm diameter using rubber molds. Ten disks of each material were prepared and subdivided into 2 groups, each containing 5 parallel samples. Osteoblasts were directly seeded onto each disk at a density of 5 × 10^4^ cells/mL. After 72 hours of incubation, samples were removed from the incubator and fixed were fixed for 4 hours at 4 °C in 4% glutaraldehyde in 0.05 M phosphate buffer (pH 7.4), dehydrated in increasing ethanol concentrations, and then critical-point-dried. They were then mounted on aluminum stubs and coated with gold-palladium (Au-Pd) (Bio-RADPolaron e5400 SEM Sputter Coating System, Kennett Square, PA, USA). Finally, samples were analyzed by SEM to elucidate the interaction cells-material.

### *In vivo* Biocompatibility

This study was conducted in accordance with the Ethical Principles adopted by the Local College of Animal Experimentation (COBEA) and was approved by Rio de Janeiro State University Ethics Committee (ID: 32/2016). The sample size estimation indicated the total amount of 9 rats per time point to obtain reliable results with a test power of 85% and statistical analysis with a significance level of 5% (p < 0.05). However, we used an addition 10% of the total sample to compensate for possible mortality or procedural errors. There were no sample losses in the present study. Thirty adult male Wistar rats (Rattus norvegicus, Albinus) weighing 250 ± 20 g, were maintained in individual stainless steel cages under a 12:12 h light-dark cycle at a controlled temperature (23 ± 2 °C) and with food and water provided *ad libitum*. Each animal received 3 sterile polyethylene tubes of 6-mm length, 1.1-mm internal diameter, (Lamedid, Osasco, Brazil). Two tubes were filled with tested materials, MTA Repair HP and White MTA, and the third tube was left empty as a negative control (control group).

After intramuscular anesthesia with ketamine Hydrochloride (25 mg/kg, Cetamin; Syntec, Cotia, SP, Brazil) associated with of Xylazine Chloridrate (10 mg/kg, Xilazin; Syntec), the backs of the animals were shaved and disinfected with 10% iodine solution (Rioquímica, São José do Rio Preto, SP, Brazil). Three 2.0 cm incisions were made a in a head-tail orientation using #15 blades (MedBlade; Medgoldman, Manaus, Brazil), 20 mm apart from each other. After subcutaneous pocketing, the tubes were implanted and skin was closed with a 4/0 silk suture (Ethicon; Johnson & Johnson, São José dos Campos, SP, Brazil). After 7, 30, and 60 days, 10 animals were killed by an anesthetic overdose.

Polyethylene tubes, with the surrounding tissues, were removed with 1-cm safety margins, fixed in 4% paraformaldehyde solution buffered at pH 7.2 with 0.1 M sodium phosphate for 48 hours. The tubes were then removed with a #11 blade (MedBlade) and the specimens were embedded in paraffin. Longitudinal 4-mm serial sections were cut parallel to the long axes of the tubes and mounted on glass slides. The histologic sections were stained with hematoxylin-eosin, Picrosirius Red, Weigert without oxidation, Gomori’s trichrome, and immunohistochemical technique for VEGF (*Vascular endothelial growth fator*) verification. High-magnification images were captured through a digital camera (DP-72; Olympus, Tokyo, Japan) coupled to a light microscope (BX 53; Olympus) and the histologic examination was carried out by a qualified pathologist, who was blinded to the type of material and implantation interval.

#### Hematoxylin-eosin

For histologic examination of the presence and type of inflammatory process, cellular proliferation, fibrosis, tissue edema, vascular congestion, occurrence of destructive process as abscess or tissue necrosis, and tissue remodeling, the sections were stained with hematoxylin-eosin solution. A semiquantitative analysis of the images was performed using the Image-Pro Plus software version 4.5.0.29 (Media Cybernetics; Rockville, MD, USA) in 10 fields per slide at magnification of 100x and 400x. The number of inflammatory cells was measured by field and was used to rate the intensity of inflammation classified in scores according to the criteria of Shahi *et al*.^33^ and following ordinal scale previously described by Cox *et al*.^34^:

Score 1 (Non-significant) = few inflammatory cells or no reaction

Score 2 (Mild) = infiltration of inflammatory cells (<25 cells) and deposits of organized collagen fibers

Score 3 (Moderate) = large infiltration of inflammatory cells (25–125 cells), limited areas of tissue edema, vascular hyperemia, and deposits of unorganized collagen fibers;

Score 4 (Severe) = very dense infiltration of acute and chronic inflammatory cells (>125 cells), areas of disseminated edema and vascular hyperemia with fibrosis, and areas of tissue destruction.

The thickness of the fibrous capsules was measured using Image-Pro Plus and were considered to be thin when thicknesses was <150 µm and thick at >150 *µ*m.

#### Picrosirius Red

For the differentiation of the collagen content (type I collagen and type III collagen), the sections were stained with a 0.1% Picrosirius-red solution and were analysed under polarized light (200x, 400x) used to observe the birefringence of the structures^26^. The birefringent collagen frequency was estimated using the following hue definition: red 2–38 and 230–256; yellow 39–51; green 52–128 and interstitial space and non-birefringent tissues 129–229. The type I collagen fibers were visualized in yellow or red, and the type III collagen fibers observed in green. The rate from each hue was calculated as a percentage of the area of each image (expressed in pixels) using ImageJ^®^ program (National Institutes of Health; Bethesda, ME, USA)^35,36^.

#### Weigert

For the observation of the fibers of the elastic system the sections were stained with a Weigert’s resorcinol fuchsin solution^37^. A qualitative analysis was performed by light microscope (100x and 200x) and the presence of elastic fibers was categorized according to the following classification: few to rare (+); moderate (++) and intense (+++).

#### Gomori’s trichrome

For the histologic examination of muscle fibers, collagen fibers and also inflammatory cells the sections were stained with Gomori’s trichrome. A complementary qualitative analysis to hematoxylin-eosin staining was performed, where the thickness and arrangement of collagen fibers, the presence of congested vessels and the presence, location and type of inflammatory process were observed under light microscopy (100x and 400x).

#### Immunohistochemistry

For the observation of the presence of angiogenesis in the tissue exposed to the inflammatory process, the sections were subjected to immunohistochemical staining for VEGF markers. The slides were dewaxed, hydrated and left in a water bath for 20 min in citrate buffer at 60 °C for antigenic recovery. They were then incubated for 30 min (20–25 °C) with 3% hydrogen peroxide to block endogenous peroxidase and washed 3x with phosphate buffered saline (PBS). Nonspecific sites were blocked using a solution with 3% PBS and BSA (bovine serum albumin) for 30 min and then incubated overnight at 4 °C with the primary VEGF antibody (diluted in 1:100). The immune reaction was revealed by a kit (HRP-DAB Reveal-Biotin, Spring, Pleaseton, CA, USA) composed of a biotinellated secondary antibody conjugated to streptavidin enzyme, and 3,3- diaminobenzidine tetrahydrochloride (DAB) as the chromogen. Negative controls were obtained by omission of the primary antibody to test the specificity of immune reaction. The immunoreaction was qualitatively classified according to the intensity of the brownish coloration in the cytoplasm of the endothelial cells, using the following scores: 0 = no staining; 1 = weak staining; 2 = medium/moderate staining and 3 = strong staining. Quantitative analysis of glycoprotein expression was classified by counting immunostained cells. The final expression of the immunoreaction was obtained by applying the modified HSCORE (histochemical score) algorithm of Jinga *et al*.^38^: HSCORE = Σ [(I + 1) × PC], where I = is the intensity of the coloration and PC = is the percentage of cells stained. The intensity of the immunostaining was then divided into: weak (+); moderate (++) and intense (+++).

### Statistical analysis

Data were submitted to statistical analysis using the SPSS® software (SPSS, Inc., Chicago, IL, USA). Parametric data of physicochemical properties and cytotoxicity assays were analyzed using Student t-test or one-way ANOVA followed by Bonferroni post-test (p < 0.05). Non-parametric data of the histopathological assays were analyzed using the Kruskal-Wallis test followed by the Dunn’s test (p < 0.05).

## Supplementary information

Physicochemical, cytotoxicity and in vivo biocompatibility of a high-plasticity calcium-silicate based material
