# Supplementary material for: Physicochemical, cytotoxicity and in vivo biocompatibility of a high-plasticity calcium-silicate based material
Source: Sci Rep. 2019 Mar 8;9:3933. doi: 10.1038/s41598-019-40365-4 (PMC6408552; doi:10.1038/s41598-019-40365-4)
Supplement: Supplementary file 1 — Physicochemical, cytotoxicity and in vivo biocompatibility of a high-plasticity calcium-silicate based material [file 41598_2019_40365_MOESM1_ESM.docx]

Supplementary Information

**Physicochemical, cytotoxicity and *in vivo* biocompatibility of a high-plasticity calcium-silicate based material**

Cláudio M. A. Ferreira^1^, Luciana M. Sassone^1^, Alexia S. Gonçalves^2^, Jorge José de Carvalho^2^, Christopher J. Tomás-Catalá^3,4^, David García-Bernal^4^, Ricardo E. Oñate-Sánchez^4^ and Francisco J. Rodríguez-Lozano^3,4*^, Emmanuel João Nogueira Leal Silva^1^

1. Endodontic Department, School of Dentistry, Rio de Janeiro State University, Rio de Janeiro, Brazil.
2. Department of Histology and Embryology, Laboratory of Ultrastructure and Tecidual Biology, Biomedical Center, Rio de Janeiro State University, Rio de Janeiro, Brazil.
3. Cell Therapy Unit at Hospital Clínico Universitario Virgen de la Arrixaca, Biomedical Research Institute of Murcia, University of Murcia, Murcia, Spain.
4. Department of Special Care in Dentistry and Gerodontology. University of Murcia, Murcia, Spain.

**CORRESPONDING AUTHOR:**

*Dr. Francisco Javier Rodríguez Lozano. DDS, PhD.

Clínica Odontológica Universitaria. H.Morales Meseguer.University of Murcia.

Av. Marqués de los Vélez, s/n, 30008

Murcia, Spain

+0034 868889518

E-mail: [fcojavier@um.es](mailto:fcojavier@um.es)

**Figure Legends**

**Figure S1.** Photomicrographs of subcutaneous tissue immunostained with VEGF after 7 days. (A) - weak intensity (arrow) of immunoreaction (brownish coloration) in the control group (empty tube); (B) - high intensity of immunostaining (arrows) in the White MTA group; (D) high intensity of immunostaining (arrows) and immunolabelled inflammatory cells (white arrows) in the MTA Repair HP group.


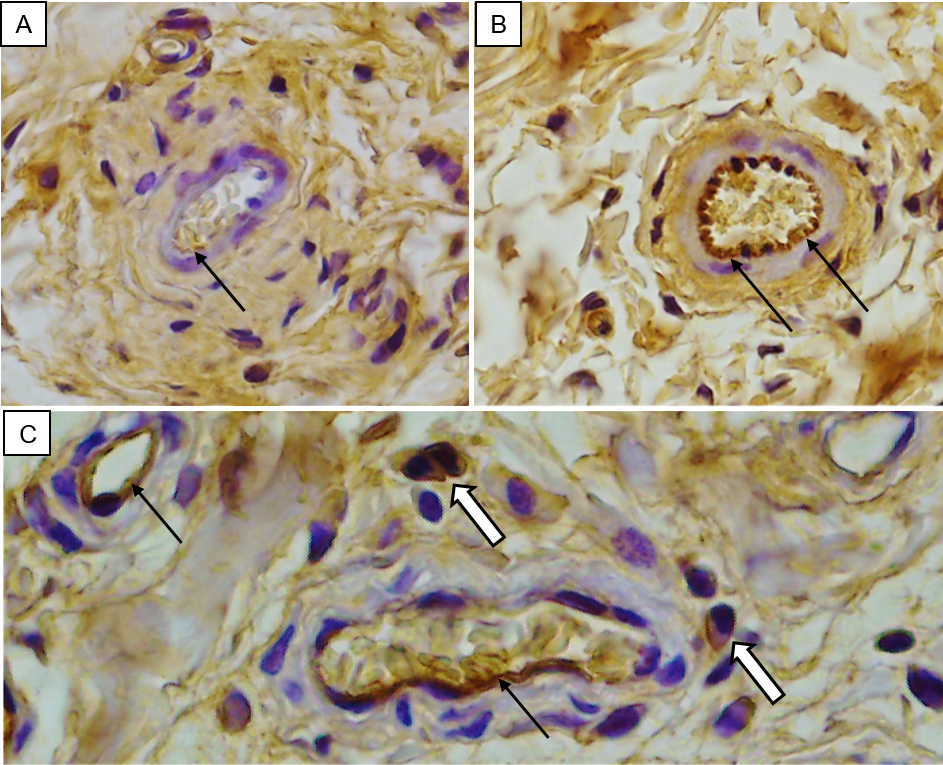


**Figure S2.** Subcutaneous tissue photomicrographs stained with Gomori’s trichrome and Weigert. (A) - White MTA group at 7 days: presence of large inflammatory infiltrate, congestive capillaries (arrows), bundles of disorganized and discontinuous collagen fibers stained in light blue. 400x. (B) - Control group at 30 days: bundles of thicker collagen fibers stained in dark blue (white arrows). 400x. (C) - White MTA group at 60 days: blood capillaries without congestion (arrows), bundles of disorganized collagen fibers stained in light blue and bundles of thicker collagen fibers stained in dark blue (white arrows). 400x. (D) – MTA Repair HP group at 7 days: presence of elastic fibers in the connective tissue close to the muscular layer (arrows), there is no elastic fibers near the fibrous capsule (white arrows). 200x

**
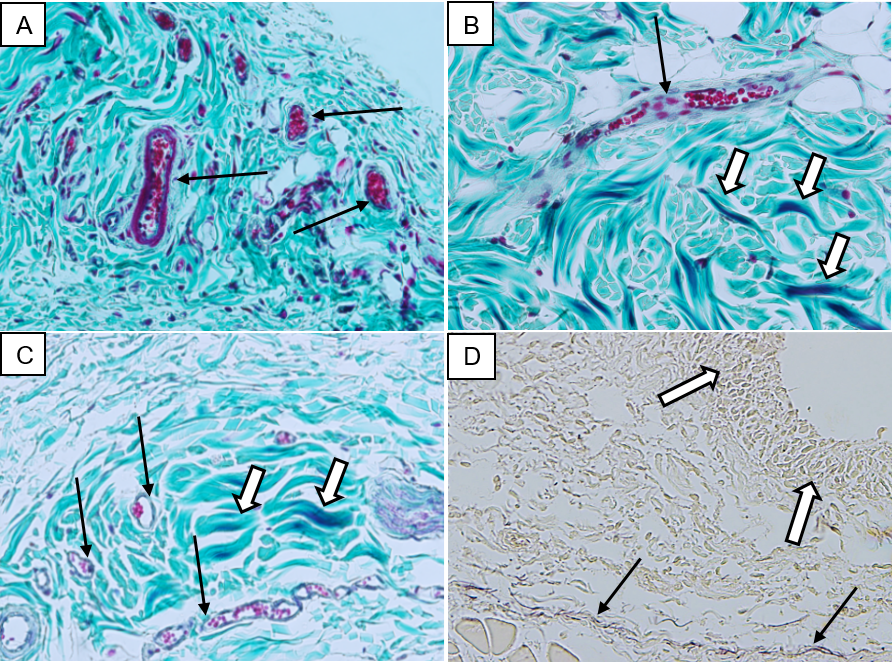
**

**Table S1.** Tested materials and their composition

| **Material** | **Composition** |
| --- | --- |
| MTA HP Repair (Angelus, Londrina, Brazil) | Tricalcium silicate (3CaO.SiO_2_);  Dicalcium silicate (2CaO.SiO_2_); Tricalcium aluminate (3CaO.Al_2_O_3_);  Calcium oxide (CaO);  Bismuth oxide (Bi_2_O_3_);  Distilled water |
| White MTA (Angelus, Londrina, Brazil) | Tricalcium silicate (3CaO.SiO_2_);  Dicalcium silicate (2CaO.SiO_2_); Tricalcium aluminate (3CaO.Al_2_O_3_);  Calcium oxide (CaO);  Calcium tungstate (CaWO_4_);  Distilled water and organic plasticizer |
